# IL‐35Ig–expressing dendritic cells induce tolerance via Arginase 1

**DOI:** 10.1111/jcmm.14215

**Published:** 2019-02-22

**Authors:** Eleonora Panfili, Giada Mondanelli, Ciriana Orabona, Roberta Bianchi, Marco Gargaro, Francesca Fallarino, Paolo Puccetti, Ursula Grohmann, Claudia Volpi, Maria Laura Belladonna

**Affiliations:** ^1^ Department of Experimental Medicine University of Perugia Perugia Italy

**Keywords:** arginase 1, dendritic cells, IDO1, IL‐35, tolerance

## Abstract

The cytokine interleukin IL‐35 is known to exert strong immunosuppressive functions. Indoleamine 2,3‐dioxygenase 1 (IDO1) and Arginase 1 (Arg1) are metabolic enzymes that, expressed by dendritic cells (DCs), contribute to immunoregulation. Here, we explored any possible link between IL‐35 and the activity of those enzymes. We transfected a single chain IL‐35Ig gene construct in murine splenic DCs (DC_35_) and assessed any IDO1 and Arg1 activities as resulting from ectopic IL‐35Ig expression, both in vitro and in vivo. Unlike *Ido1*,* Arg1* expression was induced in vitro in DC_35_, and it conferred an immunosuppressive phenotype on those cells, as revealed by a delayed‐type hypersensitivity assay. Moreover, the in vivo onset of a tolerogenic phenotype in DC_35_ was associated with the detection of CD25^+^ CD39^+^, rather than Foxp3^+^, regulatory T cells. Therefore, *Arg1*, but not *Ido1*, expression in DC_35_ appears to be an early event in IL‐35Ig–mediated immunosuppression.

## 1 INTRODUCTION

The control of immune response is operated by specialized cells, soluble molecules and membrane‐bound signals, which modulate the intensity of immune reactivity and preside over the maintenance of homoeostasis. An imbalance between immunity and tolerance mechanisms can lead to pathological conditions, such as autoimmune diseases or neoplasia, characterized by excessive or deficient control of immune reactivity respectively.

Dendritic cells (DCs) are professional antigen‐presenting cells with a key role in determining the outcome of the immune response, forcing naïve T cells into either activation or differentiation into regulatory T cells (Tregs).^1^ The components of the local microenvironment critically take advantage of the plasticity of DCs, resulting in phenotype changes. The tolerogenic molecules CTLA‐4, TGF‐β and interleukin 35 (IL‐35) are particularly effective in turning otherwise immunogenic CD8α^−^ DCs into tolerogenic cells.^2^, ^3^ Reprogramming of a cell's phenotype involves an interplay between metabolic and immunological events known as cellular immunometabolism.^4^ In CD8α^−^ DCs, the increased metabolism of specific amino acids and the subsequent production of regulatory catabolites critically contribute to the acquisition of a newly expressed suppressive phenotype. The amino acid degrading enzymes indoleamine 2,3‐dioxygenase 1 (IDO1) and arginase 1 (Arg1) are major components of immunometabolic pathways in DCs.^5^

Interleukin‐35 is a heterodimeric cytokine belonging in the IL‐12 family. It powerfully dampens immune responses by suppressing T‐cell proliferation and inducing the expansion of specific subsets of Tregs and regulatory B cells. Although elevated tissue and plasma levels of IL‐35 are associated with a poor prognosis in many malignant tumours,^6^ the cytokine has a protective role in the prevention of autoreactivity in several experimental autoimmune models and in human autoimmunity as well.^7^

IDO1 and Arg1 control tryptophan and arginine metabolism, respectively. IDO1 degrades the essential amino acid l‐tryptophan to l‐kynurenine. In T lymphocytes, l‐tryptophan depletion activates an integrated stress response triggered by GCN2, inhibiting cell proliferation and inducing anergy by down‐regulating TCR's ζ chain.^8^, ^9^ Moreover, l‐kynurenine is an endogenous agonist of the Aryl Hydrocarbon Receptor, thus promoting the expansion of Treg cells and acting to up‐regulate *Ido1* expression in a feedforward loop in DCs.^10^ Arg1 hydrolyses l‐arginine into urea and l‐ornithine, which is a substrate for ornithine decarboxylase (ODC), to produce polyamine pathway catabolites. Although l‐arginine consumption by Arg1 is a well‐known immunoregulatory mechanism at work in M2 macrophages and in myeloid‐derived suppressor cells in many tumour settings,^11^ only recently has the immunosuppressive function of polyamines been unveiled in DCs.^12^

In the current study, we investigated the possible role of IDO1 and Arg1 enzymes as potential immunometabolic effectors downstream of the tolerogenic action of IL‐35Ig in splenic CD8α^−^ DCs.

## 2 MATERIALS AND METHODS

### 2.1 Mice

Eight‐ to ten‐week‐old female C57BL/6 mice were purchased from Charles River Breeding Laboratories and *Ido1* ^−/−^ mice from the Jackson Laboratory. All in vivo studies were in compliance with National and Perugia University Animal Care and Use Committee guidelines.

### 2.2 Dendritic cell purification, transfection and treatment

Splenic DCs were fractionated using positive selection columns combined with CD11c and CD8 MicroBeads (Miltenyi Biotec, Germany).^13^ Purified CD8α^−^ DCs were transfected by DOTAP (Roche, USA) with IL‐35Ig or control Ig gene constructs^3^ and incubated overnight before in vitro analysis or in vivo administration. Nω‐hydroxy‐nor‐Arg (nor‐NOHA; Bachem, Switzerland) 150 μmol/L was added 1.5 hours before transfection.

### 2.3 Real‐time PCR and cytokine measurement

Real‐time PCR analyses for mouse *Ido1*,* Arg1* and *Gapdh* were carried out using previously reported specific primers.^12^ Values were calculated as the ratio of the specific gene to *Gapdh* expression, as determined by the relative quantification method (ΔΔCT; means ± SD of triplicate determination).^12^ Mouse TGF‐β (Affymetrix, Santa Clara, USA), IFN‐γ and IL‐4 (Thermo Fisher Scientific, USA) ELISA kits were used to measure cytokines concentrations in culture supernatants.

### 2.4 In vivo treatment, skin test assay and flow cytometry

The skin test assay has previously been described.^3^, ^14^ Briefly, purified CD8α^−^ DCs were combined with a minority fraction of the same cells (5%) transfected either with the IL‐35Ig gene construct (DC_35_) or with the Ig tail control (DC_Ig_), incubated overnight, pulsed with the HY peptide in vitro (5 μmol/L, 2 hours at 37°C), and intravenously (i.v.) transferred (3 × 10^5^ cells/mouse) into recipient hosts for the in vivo sensitization. Two weeks later, a delayed‐type hypersensitivity (DTH) response was measured to intrafootpad (i.f.p.) challenge with the eliciting peptide, and results were expressed as footpad weight increase in peptide‐injected footpad over vehicle‐injected counterparts. Alternatively, on day +14, mice were intraperitoneally (i.p.) boosted with 100 μg of HY in saline and, after 24 hours, CD25^+^, CD39^+^ and Foxp3^+^ regulatory T cells were stained in mesenteric lymph nodes (MLN), as described.^3^ Samples were analysed on LSR Fortessa (BD Biosciences, USA) flow cytometer, using FlowJo analysis software (Tree Star, USA).

### 2.5 Statistical analysis

In vitro data were analysed by unpaired Student's *t* test. In the skin test assay, paired data were evaluated by paired Student's *t* test in each group of mice, using the vehicle‐injected footpad of individual mice as an internal control.

## 3 RESULTS

### 3.1 Ectopic IL‐35Ig induces in vitro *Arg1*, but not *Ido1*, in DC_35_

The ectopic expression of IL‐35Ig, after transfection of the gene construct into murine splenic CD11c^+^CD8α^−^ DCs, was previously demonstrated to confer powerful immunosuppressive properties on those cells. The presentation of diabetogenic autoantigen IGRP by DC_35_ in prediabetic NOD mice protected animals from the occurrence of overt diabetes by a long‐lasting antigen‐specific tolerance.^3^

**Figure 1:**
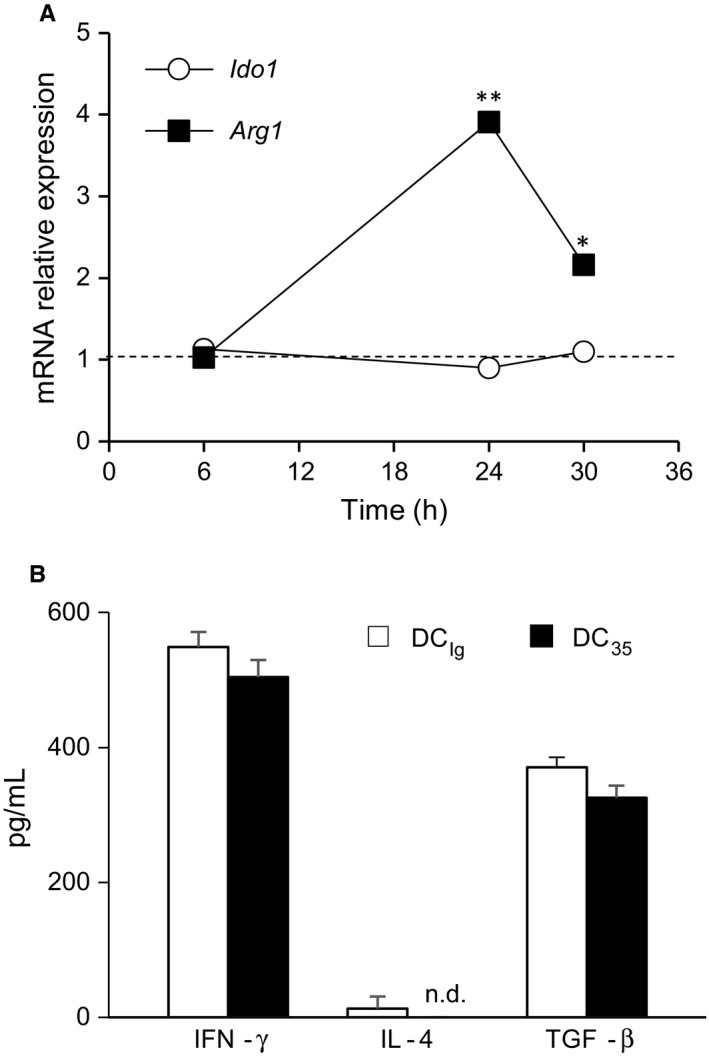
*Arg1* but not *Ido1* transcript is induced in vitro in DCs expressing ectopic IL‐35Ig. A, Real‐time PCR analysis of *Ido1* and *Arg1* transcripts in splenic DCs transfected with the IL‐35Ig single chain gene construct (DC_35_) or Ig tail control (DC_I__g_). Data (means of three experiments using triplicate samples) represent the fold change expression of *Ido1* and *Arg1* transcripts in DC_35_ normalized to the expression of *Gapdh* and reported as relative to results in DC_I__g_ for each time‐points. Dotted line denotes a fold change = 1. *P < 0.05, **P < 0.01 (Student's *t* test). B, Secretion of IFN‐γ, IL‐4 and TGF‐β in supernatants of DC_35_ or DC_I__g_ 24 h after transfection. n.d.= not detectable. Results are the mean ± SD from three different experiments (Student's *t* test).

To interrogate the effector mechanisms underlying the immunosuppressive outcome of IL‐35Ig transfection in DCs responsible for the long‐term tolerance observed in vivo,^3^ we first analysed the immunometabolic programme acquired by DC_35_ in vitro after IL‐35Ig transfection. As the increased expression of the amino acid degrading enzymes IDO1 and/or Arg1 is a critical condition for the acquisition of suppressive functions by DCs, we investigated the possible induction of the two enzymes as a consequence of IL‐35Ig ectopic expression. In a time course experiment, DC_35_ and control DC_Ig_ (i.e. transfected with Ig tag) were incubated for 6, 24 or 30 hours after transfection. Although *Ido1* expression was similar in DC_35_ and DC_Ig_ over time, *Arg1* was significantly increased in DC_35_ relative to DC_Ig_ at 24 hours (3.9‐fold) and at 30 hours (2.2‐fold) (Figure 1A). IFN‐γ, IL‐4 and TGF‐β, the most potent inducers of *Ido1*,* Arg1* or both, respectively,^12^ were not differentially secreted by DC_35_ and DC_Ig_ in culture supernatants at 24 hours post transfection (Figure 1B). Therefore, besides the mere production of a tolerogenic cytokine, DC_35_ seems to be endowed with an additional suppressive immunometabolic effector mechanism, namely, the expression of *Arg1* induced by ectopic IL‐35Ig.

### 3.2 Arg1 is required for the tolerogenic effect of DC_35_ in vivo

**Figure 2:**
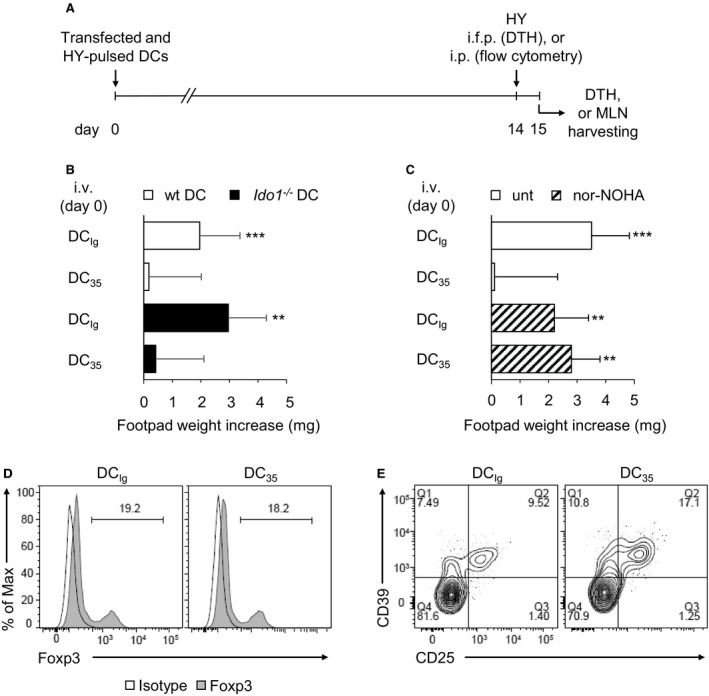
Arg1 is the effector enzyme required for the immunosuppressive action of DC_35_. A, Schematic representation of in vivo treatments. Mice were sensitized on day 0 by intravenous (i.v.) injection of HY‐loaded DC_35_ or DC_I__g_. Two weeks later, they were challenged intrafootpad (i.f.p.) for skin test assay or intraperitoneally (i.p.) boosted for flow cytometry analysis, both with HY peptide. On day +15, DTH response was recorded, or MLNs were harvested for T‐cell staining. B and C, Skin test reactivity of mice sensitized with DC_35_ or DC_I__g_. Splenic HY‐pulsed immunostimulatory CD11c^+^ CD8α^−^ DCs combined with a minority fraction (5%, indicated) of DC_35_ or control DC_I__g_ were i.v. transferred into syngeneic C57BL/6 recipient female mice to be assayed for skin reactivity to the eliciting peptide. The minority fractions were purified from either wild‐type (wt DC) or *Ido1* ^−/−^ mice (*Ido1* ^−/−^ DC) (B), and wild‐type DCs was either untreated or pretreated in vitro with the arginase inhibitor nor‐NOHA 1.5 h before transfection (C). Skin reactivity of the recipient mice (n = 6 per group) to the eliciting peptide is represented as change in weight of treated footpads vs vehicle‐receiving counterparts. Results are representative of two independent experiments (mean ± SD). Significance is referred to a two‐tailed paired Student's *t* test (experimental vs control footpads) in each group of mice. **P < 0.01; ***P < 0.001. D and E, Flow cytometry analysis of Foxp3^+^ and CD25^+^ CD39^+^ cells among CD4^+^ T‐cell population of MLN at day +15, after i.v. sensitization with HY‐loaded DC_35_ or DC_I__g_ (day 0) and i.p. boost with HY peptide (day +14) (pools of five mice per group). Isotype controls were included in the analysis and number (upper right quadrant) indicates the percentage of double‐positive cells.

To confirm the selective involvement of Arg1 (Figure 1A) in the suppressive mechanisms activated by IL‐35Ig in DC_35_ and to further verify if either of the two enzymes might act as tolerogenic effector of the cytokine, DC_35_ lacking either IDO1 (*Ido* ^−/−^ DC_35_) or Arg1 (nor‐NOHA–treated DC_35_) was assayed in vivo for their ability to inhibit antigen‐specific immune response. In DTH experiments, 2 weeks after mice sensitization with the HY‐peptide–loaded DCs, the induction of immune reactivity vs tolerance was evaluated through an intrafootpad challenge of the HY antigen, according to an established protocol^3^, ^14^ (Figure 2A). Wild‐type DC_35_ (wt DC_35_) were able to prevent the immunogenic DTH response, otherwise observed in the DC_Ig_ control group (Figure 2B). Likewise, the loss of IDO1 function in DC_35_ (*Ido* ^−/−^ DC_35_) did not modify the unresponsiveness to skin test following wt DC_35_ administration. On the contrary, Arg1 inhibition in DC_35_ by the specific catalytic inhibitor nor‐NOHA reverted the suppressive response seen with untreated DC_35_ and resulted in a significant footpad weight increase upon skin test challenge, similar to the nor‐NOHA–treated DC_Ig_ control group (Figure 2C). Therefore, skin test experiments excluded the involvement of IDO1 and rather depicted Arg1 enzyme as a relevant DC_35_ effector triggering tolerogenic mechanism in vivo.

Moreover, regulatory T‐cell populations induced in vivo by sensitization with HY‐pulsed DC_35_ and locally recalled by i.p. boost on day +14 with the same peptide were investigated by flow cytometry in MLN (Figure 2A). Interestingly, in accordance with a previous study on the protective effect of DC_35_ in autoimmune diabetes,^3^ an increased percentage (8.6%) of CD25^+^CD39^+^ T cells, rather than Foxp3^+^ T cells, was observed in DC_35_‐sensitized group relative to DC_Ig_‐sensitized group (Figure 2D and E). These data confirm that DC_35_ presented HY peptide in a tolerogenic manner and triggered a suppressive response mediated by Arg1 activation and involving CD25^+^CD39^+^, rather than Foxp3^+^, regulatory T cells.

## 4 DISCUSSION

The immunosuppressive role of IL‐35 has been observed and confirmed in many different studies,^6^ so that this member of the IL‐12 family belongs in the small group of cytokines capable of suppressing the immune response. A new aspect of IL‐35 contribution to immune regulation is the possible effect of this cytokine on the expression of amino acid degrading enzymes, and therefore on their immunosuppressive function. The *Arg1* induction we found in DC_35_ appears to be an event related to the autocrine/paracrine action of ectopic IL‐35Ig and independent from the production in culture supernatant of either IL‐4 or TGF‐β, two main inducers of *Arg1* in DCs.^12^ A potential mechanism (still to be explored) underlying the increased expression of *Arg1* in DC_35_ could be the activation of the STAT3 transcription factor, already known to be phosphorylated along the IL‐35 signalling pathway in both T and B cells^15^ and to directly bind multiple sites of the *Arg1* promoter in myeloid‐derived suppressor cells.^16^ On the contrary, *Ido1* expression resulted unaffected by IL‐35Ig in DC_35_, similar to the results in a study on monocyte‐derived DCs treated with recombinant IL‐35.^17^ However, the early induction of *Arg1* in vitro by IL‐35Ig in DC_35_ may not exclude the late involvement of IDO1 in vivo, according to the documented relay pathway between the two enzymes.^12^

The finding that Arg1 is a downstream effector of IL‐35 has immunological relevance for several aspects. In IL‐35–producing DCs (i.e., DC_35_, and most likely IL‐35^+^ DCs^18^, as well) *Arg1* induction might represent a local amplification loop of tolerance, targeting more precisely those T cells that interact with such suppressive DCs in the immunological synapsis. Moreover, the translational potential of a cell therapy with DC_35_ loaded with a specific autoimmune peptide^3^ is confirmed and reinforced by the new data of Arg1 involvement in IL‐35 tolerogenic effect. Finally, IL‐35 is emerging as an important target in tumour immunotherapy because of its inactivation could lead to the inhibition of Arg1, one of the most important immune checkpoints allowing tumour immune escape.^5^

## CONFLICTS OF INTEREST

The authors confirm that there are no conflicts of interest.
